# Parenteral versus enteral potassium suppletion in icu patients: does it make a difference?

**DOI:** 10.1186/2197-425X-3-S1-A182

**Published:** 2015-10-01

**Authors:** L Hessels, A Oude Lansink, M Hoekstra, W Bult, W M W Nijsten

**Affiliations:** Department of Critical Care, University of Groningen, University Medical Center Groningen, Groningen, Netherlands; Department of Anesthesiology, University of Groningen, University Medical Center Groningen, Groningen, Netherlands; Department of Clinical Pharmacy and Pharmacology, University of Groningen, University Medical Center Groningen, Groningen, Netherlands

## Introduction

Hypokalemia is a common electrolyte disturbance in the ICU, which makes potassiumchloride (KCl) suppletion a frequent necessity. Parenteral KCl suppletion is mostly used in critically ill patients, but it is associated with safety risks, such as overcorrection and line-infections. Enteral KCl suppletion could be a safe and effective alternative in many instances. However, it is not known whether bioavailability of enterally administered KCl is sufficient in critically ill patients. Renal potassium excretion (RPE) was used as a measure of absorbed KCl.

## Objectives

To compare the RPE in patients receiving parenteral KCl with patients receiving enteral KCl during regular potassium control in the ICU.

## Methods

This study was a prospective, observational study from February to March 2015, evaluating all patients with a minimal length of ICU-stay of 3 days in a university teaching hospital. Potassium was regulated by our validated computerized potassium regulation protocol, GRIP-II^1^. KCl-rate was advised by GRIP-II and the administration route (parenteral or enteral) was decided and recorded by an ICU nurse. KCl was never given as bolus neither intravenously nor enterally.

Based on KCl administration route, patients were assigned to either the enteral or parenteral group per day. If patients received both enteral and parenteral KCl on the same day were excluded from analysis, as were days when patients received renal replacement therapy. RPE was determined in 24-hour urines and compared between the enteral and parenteral group. The use of diuretics was recorded as well.

## Results

A total of 101 ICU patients with altogether 678 ICU days were included. In 49 (49%) patients only parenteral KCl was supplied and in 27 (27%) only enteral KCl was supplied. During 376 ICU days, potassium was administered intravenously and for 302 ICU days enterally. Mean ± SD plasma potassium was 4.1 ± 0.4 and 4.1 ± 0.4 mmol/L in these groups. The use of diuretics per day was slightly higher in the enteral group (120 enteral, 112 parenteral; p = 0.007). We collected 534 (79%) RPE measurements. RPE was 65 ± 33 for the parenteral group and 72 ± 35 mmol/d for the enteral group (p = 0.022). Upon multivariate analysis with ICU day, diuretic use, plasma potassium, plasma creatinine and mode of KCl administration as variables, mode of KCl administration was not related with RPE.

## Conclusions

Enteral KCl has a bioavailability similar to parenteral KCl in ICU patients.

On the basis of these observations we plan a RCT to assess the effect of the optimal mode of KCl administration in critically ill patients.
